# Pathophysiology and Management of Glycemic Alterations before and after Surgery for Pheochromocytoma and Paraganglioma

**DOI:** 10.3390/ijms24065153

**Published:** 2023-03-08

**Authors:** Chiara Lopez, Chiara Bima, Martina Bollati, Fabio Bioletto, Matteo Procopio, Stefano Arata, Daniele Giuseppe Candela, Guglielmo Beccuti, Ezio Ghigo, Mauro Maccario, Mirko Parasiliti-Caprino

**Affiliations:** Endocrinology, Diabetes and Metabolism, Department of Medical Sciences, University of Turin, 10126 Turin, Italy

**Keywords:** pheochromocytoma, secondary diabetes mellitus, glucose intolerance, hypoglycemia, catecholamines, paraganglioma

## Abstract

Glycemic alterations are frequent in patients with pheochromocytoma and paraganglioma (PPGL), but the real incidence of secondary diabetes mellitus (DM) is uncertain, because prospective multicenter studies on this topic are lacking in the literature. The main pathophysiological mechanisms of glucose homeostasis alterations in PPGL, related to catecholamine hypersecretion, are impaired insulin and glucagon-like peptide type 1 (GLP-1) secretion and increased insulin resistance. Moreover, it has been reported that different pathways leading to glucose intolerance may be related to the secretory phenotype of the chromaffin tumor. Predictive factors for the development of glucose intolerance in PPGL patients are a higher age at diagnosis, the need for a higher number of anti-hypertensive drugs, and the presence of secreting neoplasms. Tumor resection is strongly related to the resolution of DM in PPGL patients, with a significant improvement of glycemic control in most cases. We can hypothesize a different personalized therapeutic approach based on the secretory phenotype. The adrenergic phenotype is more closely related to reduced insulin secretion, so insulin therapy may be required. On the other hand, the noradrenergic phenotype mainly acts by increasing insulin resistance and, therefore, insulin-sensitizing antidiabetic agents can find a greater application. Regarding GLP-1 receptor agonists, the data suggest a possible promising therapeutic effect, based on the assumption that GLP-1 secretion is impaired in patients with PPGL. The principal predictors of remission of glycemic alterations after surgery for PPGL are a lower preoperative body mass index (BMI), a larger tumor, higher preoperative catecholamine levels, and a shorter duration of the disease (under three years). Otherwise, after resection of PPGL, hypoglycemia can occur as the result of an excessive rebound of preoperative hyperinsulinemia. It is a rare, but potentially severe complication reported in a lot of case reports and a few small retrospective studies. Higher 24-h urinary metanephrine levels, longer operative times and larger tumors are predictive factors for hypoglycemia in this setting. In conclusion, alterations of carbohydrate metabolism are clinically relevant manifestations of PPGL before and after surgery, but there is the need to conduct multicenter prospective studies to obtain an adequate sample size, and to allow the creation of shared strategies for the clinical management of these potentially severe manifestations of PPGL.

## 1. Introduction

The pheochromocytomas and paragangliomas (PPGLs) are rare neuroendocrine tumors arising from adrenomedullary chromaffin cells and from the autonomic paraganglia, respectively. The clinical presentation of PPGLs is extremely variable and overlaps with many other pathological conditions, representing a challenge in the diagnostic work-up. The association between PPGLs and the impairment of glucose metabolism has been described in the literature, mainly through case reports and small case series [1,2]. However, there is still an underestimation of PPGL as a secondary cause of diabetes mellitus (DM). Whereas arterial hypertension, especially if paroxysmal or resistant, is the most frequent reason for diagnosing PPGLs, the association between hypertension and DM is rarely considered a clue for the diagnosis of PPGLs. Moreover, the management of glucose intolerance in PPGL patients is often challenging: glycemic disturbances are frequently severe and difficult to treat, requiring, in some cases, insulin therapy. In fact, several case reports showed patients with PPGLs affected by treatment-resistant DM or hyperglycemic emergency in the context of newly diagnosed DM [3]. Moreover, the perioperative period is extremely critical, mainly due to hypoglycemia rebound, which occurs frequently after tumor removal and is difficult to manage. Therefore, in this review we aimed to discuss and analyze the most important evidence about the main mechanisms involved in glycemic alterations in PPGLs, and their management before and after surgery for tumor resection.

## 2. Epidemiology of Glucose Intolerance in PPGLs

A certain degree of glycemic alterations is frequent in patients affected by PPGLs, but there is a wide range in the estimated incidence of DM. In epidemiological studies, the prevalence of DM in PPGL patients was reported to vary between 21 and 37% [4,5,6,7]. The prevalence is lower (15–18%) if we consider studies without the aim of exploring the pre-test probability of glycemic disorders [2,4,8,9]. Elenkova et al. [5] conducted a retrospective study on 204 patients affected by pheochromocytoma (PCC), and found a prevalence of glycemic disorders in 49.5% of subjects. In another retrospective study performed by Beninato et al. [4], the authors investigated 153 patients with PCC which showed the presence of DM in 23% of them, with a remission of the glycemic alteration after surgery in 79% of cases. Furthermore, the incidence of hypoglycemia after surgical tumor resection must also be considered. Akiba et al. [10] showed a prevalence of severe postoperative hypoglycemia of 13%. Similarly, Araki et al. [11] demonstrated that 21% of the 49 patients who had undergone surgery for PCC experienced postoperative hypoglycemia. 

## 3. The Mechanisms of Glucose Homeostasis Impairment in PPGL

**Figure 1:**
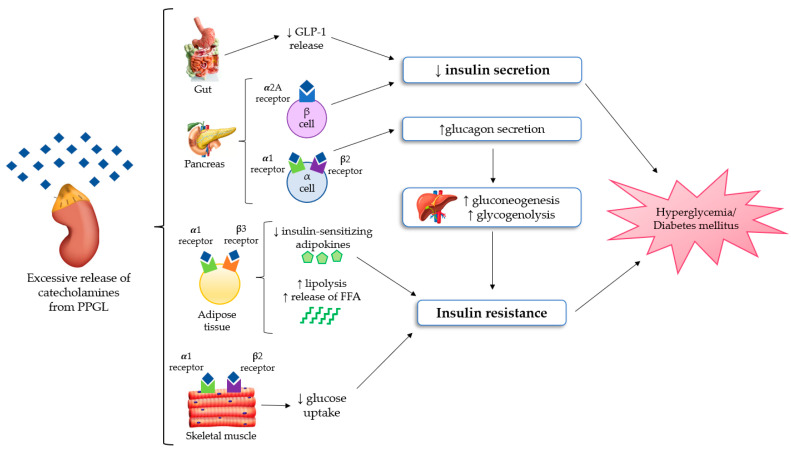
The mechanisms of glucose homeostasis impairment in PPGL [15,16,17,18,19,20,21,22,23]. Abbreviations: FFA, free fatty acid; GLP-1, Glucagon-like peptide 1; PPGL, Pheochromocytomas and paragangliomas.

The most important mechanisms regarding glucose homeostasis alterations in PPGLs are impaired insulin secretion and/or increased insulin resistance, but it is still unclear which of these factors is the most prevalent one. Several studies demonstrated that insulin secretion is compromised in PPGL as the result of the inhibitory effect of catecholamines [12,13], mainly through adrenergic α2 receptors of β-cells in pancreatic islets [14,15]. Among the different α2 receptors subtypes, the α2A ones suppress insulin secretion in pancreatic β-cells [15]. On the other side, increased insulin resistance is related to multiple mechanisms. Firstly, stimulation of gluconeogenesis and glycogenolysis in the liver, primarily due to glucagon excess. In fact, the stimulation of α1 and β2 receptors on pancreatic α-cells causes an increase in glucagon secretion [16]. Secondly, in adipocytes, the stimulation of β3 and α1 receptors increases lipolysis, and consequently the free fatty acid levels [17]. Moreover, concerning skeletal muscle, several authors showed that the stimulation of β2 and α1 receptors can stimulate glucose uptake in myocytes [18,19]. Although PPGLs are well known to be associated with glucose intolerance, there are limited studies available that explore the effect on insulin secretion and sensitivity. In a retrospective study on 13 patients with PPGLs, Komada et al. [20] documented that chromaffin tumors could impair the insulin secretory response, especially in the first phase of secretion. The authors highlighted that insulin secretion, evaluated both through the hyperglycemic clamp and oral glucose tolerance test (OGTT), significantly improved after surgery. Instead, the results concerning insulin resistance were controversial because changes of the insulin sensitivity index on hyperinsulinemic–euglycemic clamps were not significant, but the authors found an improvement in the homeostasis model assessment of insulin resistance (HOMA-IR) from the preoperative to postoperative period. Petrack et al. [21] conducted a prospective study on 18 patients with PCC to explore the contribution of the incretin system in the pathogenesis of dysglycemia in PPGL. They demonstrated impaired insulin secretion and glucose intolerance in patients with PCC using the meal test and the homeostasis model assessment of β-cell function (HOMA-β). Furthermore, they suggested that compromised insulin secretion could be related to impaired glucagon-like peptide 1 (GLP-1) secretion, with an increase in glucagon levels, probably due to the lack of GLP-1 suppression of glucagon production. Moreover, Diamanti-Kandarakis et al. [22] evaluated glucose intolerance in five patients with PCC using OGTT and the euglycemic clamp technique. They documented an improvement of glucose intolerance and a reduction of insulin resistance after surgery. On the contrary, the administration of non-selective adrenergic α-blockers and β-blockers without the surgical removal of the lesion may induce an improvement of glucose alterations, which is usually less relevant in comparison to that obtained after tumor resection. Furthermore, some data suggested a possible relationship between PPGL-related impaired glucose metabolism and adiponectin, an adipocytokine with an anti-diabetic, anti-inflammatory and anti-atherogenic action. A study conducted by Elenkova et al. [23] aimed to evaluate the correlation between adiponectin levels and insulin resistance in patients with endocrine hypertension due to PCC, compared with patients affected by essential hypertension and control healthy subjects. The authors highlighted that adiponectin levels were lower in patients with PCC and which increased after tumor surgical removal. There was a negative correlation between adiponectin serum concentrations and preprandial glucose, insulin levels and HOMA as a marker of insulin sensitivity. Interestingly, these data suggested that hypoadiponectinemia in PCC patients could possibly be another pathogenic factor of glucose intolerance. All the pathophysiological mechanisms of glucose homeostasis impairment in PPGL are shown in Figure 1. 

## 4. The Differences in Glucose Intolerance According to Secretory Phenotype

According to the different affinities of epinephrine and norepinephrine on adrenergic receptors and their different effects on glucose metabolism, it is likely that the tumor secretory phenotype could differently affect glucose metabolism in PPGL patients. Recently, Abe et al. [24] conducted a study on 12 patients who had undergone surgery for PCC, to explain the mechanisms of glucose intolerance about the type of hormone secretion. The authors investigated the association between the changes of urine metanephrine and normetanephrine (epinephrine and norepinephrine metabolites, respectively) and those of HOMA-β/HOMA-IR from the preoperative to the postoperative period. The study showed that an excess of epinephrine can affect glucose metabolism mainly through impaired insulin secretion, instead of an excess of norepinephrine which can have a prevalent effect in terms of increased insulin resistance. These results could be explained by the molecular mechanisms of adrenergic receptors affinities. In fact, epinephrine has a higher affinity for adrenergic α2 receptors, which suppress insulin secretion in pancreatic β-cells; conversely, norepinephrine has a higher affinity for α1 receptors that are related to the pathway of insulin resistance through increased free fatty acid, glucagon secretion and glucose uptake in muscle. In a similar way, there could also be a difference in GLP-1 secretion according to the tumor secretory profile. An “in vivo study” documented that norepinephrine could inhibit GLP-1 secretion through adrenergic α1 and/or α2 receptors [25]; instead, a recent “in vitro” study showed that epinephrine could increase GLP-1 secretion through adrenergic α1, α2, and β1 receptors [26]. Therefore, future studies could elucidate the association between GLP-1 and type of catecholamine secretion in patients with PPGL. 

## 5. Predictive Factors of Development of Glucose Intolerance in PPGL

According to the 2020 Consensus of the European Society of Hypertension [27], the presence of hypertension and DM in patients under 50 years of age with a normal body weight (Body Mass Index—BMI < 25 Kg/m^2^) represents a strong suspicion of PPGL, and biochemical testing is therefore recommended. This indication represents a novelty in comparison with the statements of the 2014 Endocrine Society Guidelines [28]. A recent Indian prospective cohort study enrolled a total of 47 patients with a suspected diagnosis of PPGL. It showed that the predictors of preoperative DM were higher with age, the need for a higher number of anti-hypertensive drugs, and the presence of a secretory tumor. Moreover, female patients and those with higher urine values of vanillylmandelic acid presented a slightly increased risk of DM, without reaching statistical significance. On the contrary, BMI was not directly associated with an increased risk of DM in this cohort of PPGL patients [29]. A retrospective study of 204 patients with histologically confirmed PPGL diagnosis highlighted that patients with a DM or prediabetes status preoperatively had a significantly higher age at diagnosis, as well as higher 24-h urine metanephrine and normetanephrine levels. Despite this, authors did not find a significant correlation between the levels of these metabolites and glycated hemoglobin (HbA1c), probably in consideration of the fact that several subjects were already being treated with antidiabetic drugs. A moderate positive correlation was revealed between age and fasting plasma glucose. The major limitation for this retrospective study was the impossibility to distinguish between pre-existing T2DM from the new onset of secondary PPGL-associated DM [5]. Krumeich et al., in a retrospective cohort study, analyzed 360 patients with PCC/PGL, finding that norepinephrine and normetanephrine were inversely associated with weight and directly associated with HbA1c at the multivariate analysis. After tumor resection, the decline of normetanephrine levels was related to the improvement of HbA1c, despite an increase in body weight. Moreover, persistently elevated catecholamines and decreasing weight were observed in metastatic or recurrent disease [30].

## 6. Predictive Factors of Remission of Glucose Intolerance after Resection of PPGL

In a retrospective study, Elenkova et al. showed that patients with persistent DM/prediabetes after surgery for PPGL had a higher BMI (before and after operation), compared to those with postoperative normalization of carbohydrate metabolism. In the same study, subjects with adequate glycemic control following only diet and/or an oral anti-diabetic monotherapy were more likely to have complete remission of DM after resection of PPGL [5]. Another retrospective study on 153 PPGL patients that underwent surgery confirmed that subjects with a lower BMI were more likely to have complete resolution of DM. In addition, patients with a larger tumor and greater preoperative variability in catecholamine levels were more likely to normalize glycemic alterations, but these relationships did not reach statistical significance. The authors demonstrated that an elevated BMI was an independent risk factor for the persistence of glucose intolerance after surgery for PPGL. Consistent with the fact that patients with a higher BMI are more likely to develop DM independently from PPGL, these subjects were more likely to continue to require treatment for diabetes, even after the source of catecholamine excess was removed. Similarly, although without reaching statistical significance, patients who resolved their DM were younger, had larger tumors, and higher preoperative hormone values. This is likely because, in these cases, the tumor is the main cause of DM onset, rather than other traditional risk factors [4].

Finally, the aforementioned Indian prospective study highlighted that lower HbA1c levels and a shorter duration of disease (less than three years) were predictive factors for DM remission. A higher age and higher preoperative BMI showed trends towards a persistent increase of DM after surgery, but without reaching statistical significance [29]. 

These data were confirmed by another Chinese retrospective study on 185 PCC patients who underwent adrenal surgery, including 67 subjects with preoperative DM. The BMI of patients with partial or complete resolution of DM after surgery was significantly lower in comparison with individuals who had no resolution. Subjects with higher preoperative catecholamine level variability and larger tumors were also more likely to achieve DM remission, but these relationships were not statistically significant [31].

## 7. Treatment of Glucose Intolerance in PPGL

According to the complex mechanisms underlying glucose metabolism impairment in PPGL, primarily related to multiple catecholamine actions, many patients with PPGL encounter difficulties in maintaining glycemic control. As shown in a study performed by Diamanti-Kandarakis et al. [22], the use of α-adrenergic or β-adrenergic blockers does not result in a significant improvement of hyperglycemia. Conversely, as previously explained, tumor resection is strongly related to the resolution of DM in PPGL patients, with a significant improvement of glycemic control in a high percentage of cases [2,4,6,31]. In the management of glycemic disturbances, we have to distinguish between treatment of hyperglycemic emergencies, such as diabetic ketoacidosis or hyperglycemic hyperosmolar states, and management of less severe conditions of DM. Treatment of diabetic emergencies must follow the rules of the management of hyperglycemic crisis, involving correction of dehydration and lowering glucose levels with careful monitoring of serum osmolarity and potassium levels. Therefore, the mainstay of treatment is represented by fluid and electrolyte replacement and insulin therapy. In this specific setting, we also emphasize that PPGL resection is frequently associated with postoperative hypoglycemia, so a slow titration of insulin dosage may be needed, and a very close perioperative and postoperative patient monitoring is mandatory. Instead, concerning treatment of PPGL-related DM, apart from diabetic emergencies, several antidiabetic agents may be used. Literature data about treatment strategies in this setting are scarce, mainly based on small case series [32]. Nevertheless, some therapeutical considerations could be raised considering the above-mentioned pathophysiological mechanisms. Metformin is currently the first-line drug treatment for T2DM. In PPGL patients, DM is strongly related to increased insulin resistance, so metformin, through its well-known effect in improving insulin sensitivity, could be effectively used in this setting. Some case series reported that patients, affected by PCC and poorly controlled DM despite high insulin doses, showed a significant improvement of glycemic imbalance after surgery. In these cases, patients were postoperatively transitioned to metformin therapy with discontinuation of insulin therapy, reaching good glycemic control [32]. However, if a significant impairment of insulin secretion is present, insulin therapy may be needed, leading to the risk of hypoglycemia. This complication could be a major concern in PPGL patients that already have a high risk of postoperative hypoglycemia. Drugs acting on incretin systems, mainly GLP-1 receptors agonists (GLP-1 RA), could also be a promising treatment option. In fact, as already explained, literature data shows that the general suppression of glucoregulatory and gut hormones is a significant contributor to impaired glucose metabolism in PPGL patients [21]. These findings could offer an interesting and safe potential therapeutic strategy, especially for treatment of patients affected by PCC with adrenergic phenotype, in the preoperative period or in the postoperative phase in case of a lack of surgical cure. Apart from this mechanism, GLP1-RA could be very important for their protective cardiovascular effects, considering that PPGL patients are at high risk of cardiovascular complications and events. Dipeptidyl peptidase 4 (DPP4) inhibitors, through their action on incretin systems, could be another interesting therapeutic option, despite the lack of evidence of a potential cardiovascular protective role. For this purpose, no data are available concerning the use of sodium–glucose cotransporter 2 inhibitors (SGLT2-I) in this clinical setting. From a pathophysiological point of view, this treatment could be useful given their well-known cardiovascular benefits, even in the prevention and treatment of heart failure. The main alert is represented by the risk of euglycemic diabetic ketoacidosis, which is increased in case of dehydration, infections, surgery, insulin dose reduction or withdrawal. 

We can also make some considerations regarding antidiabetic agents that are less frequently used in clinical practice. Acarbose, the most widely used alpha glucosidase inhibitor, is an oral antidiabetic drug that mainly acts by delaying the rate of carbohydrate digestion and absorption. However, based on its possible role as an indirect non-nutritional GLP1 secretagogue, this therapeutical option may be considered in this clinical context. Sulfonylureas and glinides belong to an old drug class of extremely limited use because of their risk of hypoglycemia and of worsening cardiovascular disease. Therefore, we do not believe that they should have a therapeutic role in PPGL-related DM. 

Glitazones are a potent insulin sensitizer, with well-documented beneficial effects on the cardiovascular risk factors associated with insulin resistance. Concerning the safety issue, the most important concern seems to be its correlation with a worsening of congestive heart failure. This is extremely important in PPGL patients for their very high risk of sudden cardiovascular complications. Conversely, we can hypothesize that drug-related fluid retention could contribute to hemodynamic stability in the postoperative period. Nevertheless, we do not think that glitazones should be adopted for treating this kind of secondary DM.

According to the differences in the actions on glucose intolerance among some types of catecholamines excess [24], we can suggest a different personalized therapeutic approach based on the secretory phenotype. Concerning glycemic disturbances, we can distinguish between the adrenergic phenotype, characterized by increased adrenaline levels regardless of noradrenalin levels, and the noradrenergic phenotype, with an isolated noradrenalin overproduction. Literature data showed that DM in PPGL is mainly related to plasma adrenalin concentrations [2]. An adrenergic phenotype is strongly related to glycemic imbalance. In this context, in case of a predominant reduced endogenous insulin secretion, insulin therapy may be required. Nevertheless, some data also suggested that in PCC with adrenergic phenotype GLP-1 secretion is typically impaired, so drugs acting on incretin systems, both GLP-1 RA and DPP4 inhibitors, could have a possible promising therapeutic effect [21]. On the other hand, the noradrenergic phenotype mainly acts by increasing insulin resistance. Therefore, in this clinical context, insulin-sensitizing antidiabetic agents such as metformin or incretin-based drugs may find a greater application [21].

## 8. Pathophysiological Mechanisms of Hypoglycemia after Surgery for PPGL

Hypoglycemia after resection of PPGL is a rare, poorly understood, but potentially severe complication, reported in a lot of case reports and a few small retrospective studies [8,33,34]. Moreover, in the early postoperative period, classic symptoms of hypoglycemia such as anxiety, sweating, chills, irritability, lightheadedness and nausea, may be misinterpreted with the residual effects of anesthesia, opioid, or β-adrenoceptor blocker assumption. If untreated, severe hypoglycemia can result in neuronal cell death and persistent neurological damage [35,36]. The most convincing pathophysiological mechanism of post-excisional hypoglycemia is the excessive rebound of hyperinsulinemia because of PCC resection [8,11]. As explained previously, patients with PPGL have glucose intolerance, which can be mediated by hypersecretion of catecholamine. This leads to increased liver glycogenolysis, the inhibition of insulin secretion from pancreatic β-cells, and the reduction of glucose uptake in the adipose and skeletal muscle cells (insulin resistance). These pathological changes can lead to preoperative hyperglycaemia. Following the excision of PPGL, a sudden drop in the values of circulating catecholamines can determine hypoglycemia in the immediate postoperative period [36]. Moreover, preoperative use of β-adrenergic blockers can lead to increased insulin production from pancreatic cells and to impair liver gluconeogenesis and the glucagon secretion mechanism, subsequently contributing to the development of hypoglycemia [33]. Sagalowsky et al. suggested that phentolamine, a non-selective α-blocker, can also contribute to hypoglycemia after resection of PPGL, even if the evidence of hypoglycemia with α-blockade is not documented [34].

**Figure 2:**
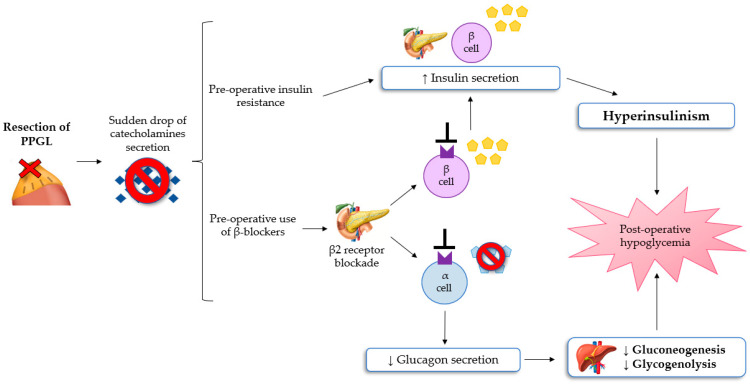
The mechanisms of hypoglycemia after surgery for PPGL [8,11,32,33]. Abbreviations: PPGL, Pheochromocytomas and paragangliomas.

Araki et al. showed how the risk of hypoglycemia is similar following the excision of PCC or paraganglioma [11]. Moreover, severe hypoglycemia appears to be more prevalent in patients with von Hippel Lindau syndrome-associated PPGL. In conclusion, it follows that all patients undergoing resection of a chromaffin tumor require intensive monitoring of serum glucose levels during and after surgery to early detect hypoglycemia [10,11]. The pathophysiological mechanism of the development of hypoglycemia after surgery for PPGL are summarized in Figure 2.

## 9. Pathophysiological Mechanisms of Hypoglycemia Related to Metastatic PPGL

In literature, there are a few case reports of hypoglycemia in patients with metastatic PPGL. The principal mechanisms proposed are subsequently:

(1) Direct glucose consumption by the tumor in the presence of an elevated burden of disease, according to the results of the 18F-FDG-PET/CT scan findings. This was reported in a few case reports, with the presence of FDG uptake predominantly in the tumor mass, and the reduction in the normal FDG uptake throughout the rest of the body, most prominently in the brain [37,38];
(2) Excessive ectopic cancer production of aberrant IGF-II or pro-IGF-II, that are less protein-bound and subsequently more bioactive than normal IGF-II. Multiple actions of these compounds can be responsible for the development of hypoglycemia, primarily in the reduction of glucose hepatic output, by decreasing hepatic gluconeogenesis and glycogenolysis. On the other hand, abnormal activation of IGF-II of IGF-1 receptors on the pancreatic alpha cells leads to suppression of the production of the insular counter-regulation hormones like GH, IGF-1 and glucagon [38];
(3) Direct ectopic release of insulin from PGL. Paraganglioma originates from neural cresta cells, and the cosecretion of insulin and catecholamines has been demonstrated by a single case report. Immunohistochemical insulin staining was detected in the neoplastic cells at the histopathological diagnosis after resection of the tumor [39];
(4) Hemorrhagic infarction of the tumor mass with subsequent cancer cells necrosis. This induces hypoglycemia with the same mechanisms that occurs postoperatively in patients undergoing adrenalectomy for PCC, due to the sudden failure of the conditions that determine hyperglycemia, predominantly the drop of plasmatic catecholamine levels [40].

## 10. Predictive Factors of Hypoglycemia after Resection of PPGL

A single-center retrospective study on 49 PPGL patients who underwent surgery showed how the incidence of hypoglycemia was not statistically different between subjects with adrenal and extra-adrenal tumors. During the preoperative oral glucose tolerance test, no significant difference was found in the immunoreactive insulin/glucose ratio between patients with and without hypoglycemia after surgery. On the contrary, higher intraoperative immunoreactive insulin/glucose ratio and 24-h urine epinephrine levels, but not 24-h urine norepinephrine levels, were predictive factors for post-adrenalectomy hypoglycemia [11]. Another retrospective study by Chen et al., with a larger sample size of 213 subjects undergoing resection of PCC, highlighted that patients who developed hypoglycemia were more likely to have higher preoperative 24-h urine metanephrine levels, experience a longer operative time, and present a larger tumor. These can therefore be considered as predictive factors for the development of postoperative hypoglycemia. Patients who experienced postoperative hypoglycemia often needed a more intensive level of care, even if no difference in the duration of hospitalization was eventually observed [8].

## 11. Treatment of Hypoglycemia Related to PPGL Resection

The first treatment of hypoglycemia is prevention, limiting as much as possible the adoption of drugs with potential hypoglycaemic risk for the therapy of preoperative secondary DM/hyperglycaemia. Symptomatic treatment considerations for hypoglycemia include oral administration of rapidly absorbed carbohydrates to provide at least 15–50 g of glucose. Moreover, if the patient is unconscious or unable to tolerate oral intake, treatment with dextrose 5–10 or 33% of continuous infusion should be started and titrated to a glycemia goal of >100 mg/dL. In emergency situations, where treatment with oral or intravenous dextrose is not feasible/available, administration of 1 mg intramuscular glucagon should be considered [36].

In consideration of the state of hyperinsulinism underlying hypoglycemia post-excision of PPGL, in patients who cannot be weaned from dextrose infusion and with the recurrence of hypoglycemia, treatment with diazoxide could be considered by virtue of the pathogenetic mechanism of this type of hypoglycemia resulting from hyperinsulinism [41]. Diazoxide inhibits insulin secretion by opening the ATP-dependent potassium channel in pancreatic β cells. Treatment is highly effective, as about 60% of patients with hyperinsulinemic hypoglycemia become symptom free. This treatment is usually suggested through fractioned doses ranging from 100 to 600 mg per day, and the most common dosage used is 100 mg tid. Side effects like fluid retention and hirsutism in women are common, but are mild and not generally alarming [42]. Besides pharmacological treatment, these subjects should follow a fractioned diet based on complex carbohydrates with slow absorption. 

Finally, in the case of refractory hypoglycemia, treatment with glucocorticoids can be used because it induces hyperglycaemia by inhibiting insulin release and increasing peripheral insulin resistance. This therapy, however, is associated with a series of well-known metabolic side effects, therefore it should be as short as possible.

Instead, in cases of metastatic PPGL-related hypoglycemia, treatment with somatostatin analogues can be considered, though off-label. They exploit the inhibitory effect on insulin secretion in the presence of specific receptors, after the evaluation with 68Ga-labeled DOTA peptide CT/PET [41].

## 12. Conclusions

A certain degree of glucose intolerance up to the development of secondary DM is frequent in patients with diagnosis of PPGL. This association has been widely described in literature, although mainly through case reports and small retrospective case series [1,2]. Similarly, the incidence of hypoglycemia after surgery for PPGL is also relevant [10,11]. If it is not adequately recognized and treated, it can lead to neuronal cell death and potential persistent neurological damage [35]. Therefore, since alterations of carbohydrate metabolism are clinically relevant manifestations, there is a need to conduct large multi-center prospective studies to improve knowledge and create shared strategies for the diagnostic and therapeutic management of PPGL-related glycemic dysregulations, which can be potentially severe and even fatal.

## Data Availability

No new data were created or analyzed in this study. Data sharing is not applicable to this article.
